# Visualizing and quantifying movement from pre-recorded videos: The spectral time-lapse (STL) algorithm

**DOI:** 10.12688/f1000research.3-19.v1

**Published:** 2014-01-21

**Authors:** Christopher R Madan, Marcia L Spetch

**Affiliations:** 1 Department of Psychology, University of Alberta, Edmonton, Alberta T6G 2E9, Canada

## Abstract

When studying animal behaviour within an open environment, movement-related data are often important for behavioural analyses. Therefore, simple and efficient techniques are needed to present and analyze the data of such movements. However, it is challenging to present both spatial and temporal information of movements within a two-dimensional image representation. To address this challenge, we developed the spectral time-lapse (STL) algorithm that re-codes an animal’s position at every time point with a time-specific color, and overlays it with a reference frame of the video, to produce a summary image. We additionally incorporated automated motion tracking, such that the animal’s position can be extracted and summary statistics such as path length and duration can be calculated, as well as instantaneous velocity and acceleration. Here we describe the STL algorithm and offer a freely available MATLAB toolbox that implements the algorithm and allows for a large degree of end-user control and flexibility.

## Introduction

**Figure 1.**
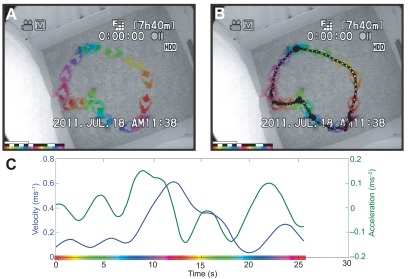
Visualizing and quantifying movement data from a single trial of a pigeon navigating an arena with four food cups. ( **A**) Spectral time-lapse (STL) image of the trial, sampled at 1 pps. First bar in bottom left corresponds to 10 seconds; second bar illustrates which frames highly overlapped with adjacent frames; third bar shows time-color mapping used. ( **B**) Path overlaid on the STL image, sampled at 6 pps. ( **C**) Velocity-acceleration plot of same movement data.

Studies of animal behaviour in open environments yield rich datasets. While behaviour can often be summarized through simple measurements (e.g., first target approached within an array, sequence of targets approached, timings of these behaviours), these measures are not always sufficient. A widely-used solution to this problem was introduced three decades ago, with a methods paper describing the use of video recordings to study animal behaviour ( Godden & Graham, 1983). Although some researchers use commercial tracking equipment, movements are sometimes recorded using standard video cameras without markers on the animal and the data are manually scored. Using simple pre-recorded video recordings, we sought to summarize both spatial and temporal information of movements within a two-dimensional image representation. Specifically, we developed spectral time-lapse (STL) images that code the animal’s position with a time-specific color and overlay them on a frame of the video to produce a summary image ( Figure 1A). We also incorporate automated tracking of the animal’s path and provide summary statistics ( Figure 1B), as well as plotting velocity and acceleration over time ( Figure 1C). Here, we describe the algorithm and offer a MATLAB toolbox that implements it, while allowing for substantial end-user control.

The challenge of visualizing movements within a two-dimensional image is not new. Although many solutions have been discussed ( Jensenius, 2012, 2013), none integrate both spatial and temporal information to sufficiently characterize a path within a single image. Time-lapse images (illustrated in Jensenius, 2013, Figure 1) concatenate a series of still images adjacently, and do not present the images within the same spatial frame. Motion history and motion average images (illustrated in Jensenius, 2013, Figure 4–Figure 7) show movements within the same spatial frame, but lose temporal information. Our solution was to color images of the target using a time-specific color, and overlay these on the background, see Figure 1A.

Our second goal was to obtain path data, specifically x- and y-coordinates of the animal at each time point. While solutions for this purpose already exist, many have drawbacks. EthoVision ( Noldus *et al.*, 2001, 2002; Spink *et al.*, 2001), a widely used movement-tracking software package, needs to be adjusted for each set-up (e.g., animal to track and type of arena). Other methodological drawbacks include requiring markers on the animal during video acquisition (e.g., Chen *et al.*, 2008), specification of templates of the animal’s shape (e.g., Kalafatić, 2003; Xu *et al.*, 2009), or the ability to only process low-resolution videos (reducing precision; e.g., Crispim Junior *et al.*, 2012). Although solutions exist that do not have these limitations (e.g., Khan *et al.*, 2006; Perner, 2001; Tort *et al.*, 2006; Tweed & Calway, 2002), our implementation of the STL toolbox in MATLAB allows the end-user to easily extract path data within the MATLAB environment (e.g., Figure 1B). To glean additional information from the path, we also calculate instantaneous velocity and acceleration (see Figure 1C).

## Materials and methods

Animal research was conducted in accordance with Canadian Council on Animal Care guidelines and with approval from the University of Alberta Animal Welfare Policy Committee. Pigeons (Columba livia) were kept on a 12:12 h light:dark cycle with light onset at 6 AM. Birds were housed individually in metal cages and kept at 85% of their free feeding weight on a diet of Kee Tee pigeon pellets and vitamin supplement. Water and grit were available ad libitum.

Here we present a spectral time-lapse (STL) image and describe the algorithm used to create the image. Figure 1A illustrates a single trial of a pigeon ( *Columba livia*) entering an arena, moving to and eating from four food cups, and returning to the starting box. The STL image allows the researcher to observe the behaviour (e.g., sequence of cups visited, efficiency of path taken) without needing to watch the video. This is particularly useful as videos are often longer in duration than the movement; in this particular trial, the raw video lasts 45 sec., while the pigeon is only visible for 25 sec. The STL image in Figure 1A was generated to show one position-per-second (pps), in other words, one colored position (i.e., pigeon) is plotted for each second. The raw video for this particular trial is included as Data File 1.

Video data was acquired using a standard video camera connected to a PC running Microsoft Windows 7 (Redmond, WA) and recorded as a MPEG-2 transport stream file using the WinTV hardware and software package (Hauppauge Computer Works Inc., Hauppauge, NY). (Note: It is not necessary for the STL method that the videos be recorded with WinTV or that the videos be saved as MPEG-2 transport stream files, this was just how we chose to digitize our video recordings). We converted the video to an uncompressed AVI format using MPEG Streamclip (Squared 5 S.R.L., Rome, Italy), but other software could be used as well. These uncompressed AVI files can be read directly into the STL toolbox.

Data File 1. Raw video used in Figures 1 and 2 Video shows a single trial of a pigeon (Columba livia) entering an arena, moving to and eating from four food cups, and returning to the starting box.

## The STL algorithm

**Figure 2.**
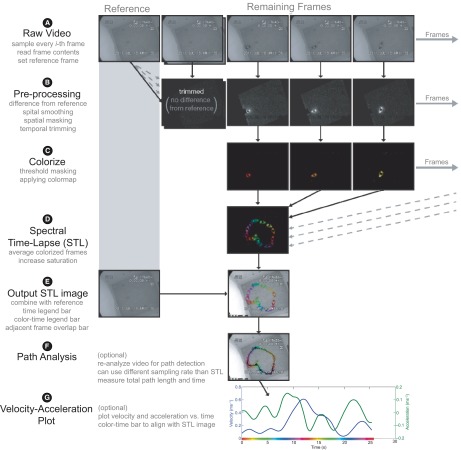
Illustration of the STL algorithm, the component stages, and examples of images at each stage. ( **A**) Loading the raw video. ( **B**) Pre-processing. ( **C**) Colorizing the frames. ( **D**) Creating the STL image. ( **E**) Outputting the STL image. ( **F**) Path analysis method. ( **G**) Velocity-acceleration plot.

The steps comprising the STL algorithm are illustrated in Figure 2. Settings that can easily be adjusted by the end user are noted in parentheses and italicized throughout. These names refer to the variable names within the STL toolbox and are found within the configuration file (config.m, see Supplementary Materials).

### A. Loading the raw video

The raw video file is read in and only every *i*-th frame is sampled ( *sampling*), as video is often acquired at higher rates than needed for the STL image. For instance, the animal’s position might be sampled at 1 pps, whereas video cameras often record at 24 or 30 frames-per-second (fps). If the original video speed has been adjusted, such as videos originally from a high-speed camera, then this can be accommodated and calculations adjusted ( *videospeed*). The STL toolbox reports the video’s original acquired fps and the STL’s pps. The sampled video frames are converted to grey-scale, as color will be used to code for time. The folder containing the raw video must be specified in the configuration file ( *path_raw*).

To allow the STL images to be based on only a portion of a video, start and end frames can be specified, ( *startFrame*, *endFrame*). An additional MATLAB function called showFrameK is included to facilitate in determining start and end frames.

In this stage, the reference frame is also defined, which is often either the first or last frame of the video, or a ‘moving average’ ( *refFrame*). The reference will be subtracted from all other frames to isolate the target animal, i.e., the change in the video frame, in the next stage. A moving average is useful when the background changes over time (e.g., lighting, bedding materials; *refSmooth*).

### B. Pre-processing

The STL algorithm implements a pre-processing stage to isolate movement data and reduce noise. Here, five pre-processing calculations were done for each frame:

First, the reference is subtracted from the given frame, to isolate changes in the frame that corresponds to the target.

Second, the difference image is spatially smoothed to reduce noise. This is implemented by convolving a two-dimensional Gaussian kernel with the given frame. Ideally, the user will calibrate the kernel size to the image, based on the animal’s size, as viewed by the camera, and video resolution ( *smooth*).

Third, if the animal is lighter colored than the background, intensity values are negative. To produce consistent color mapping in the next stage, we reverse these values so that intensity of the target is always positive.

Fourth, irrelevant portions of the frame are masked out to improve the signal-to-noise ratio and later target detection. Two approaches are used to do this, a pre-made static mask ( *doMask*) and a dynamic detection of an overlay ( *cleanWhite*). For the pre-made mask, the filename to the mask image must be provided ( *maskName*). For the overlay, any pixels with an intensity value above a set threshold are ignored ( *white*). This is useful if a timestamp or other overlay is hard-coded into the video, as in Figure 1A.

Fifth, we trim frames from the start and end of the video that did not contain the target; this feature can be disabled by the end user ( *disableTrim*). Frames are only retained if they are sufficiently different from the reference, based on thresholds ( *threshMask, threshTrim*). At this point, only frames containing temporal information about the movement are retained.

Figure 2 shows example images of the frames after these calculations.

### C. Colorizing the frames

A mapping of time-to-color is created for each of the retained frames. This mapping is adjustable, but usually corresponds to one or two color cycles ( *cmap*). A mask is then created such that only pixel intensities that surpass a threshold are retained ( *threshMask*), further removing noise. At this point, the spatial information corresponding to the target has been isolated. The color specific to the given frame is then applied, see Figure 2.

### D. Creating a spectral time-lapse (STL) image

All colorized frames are averaged to produce a single frame that is essentially the STL image. To improve color visibility after averaging, the saturation of the averaged frame is amplified ( *oversatCol*).

### E. Outputting the spectral time-lapse (STL) image

To produce the final STL image, we overlay the averaged frame on the reference ( *refFrame*). To further improve visibility of the colors, we increase the saturation of the reference ( *oversatRef*). Legend bars are added to the image to show (a) actual time, (b) indicate overlapping frames as would occur if the target pauses, and (c) time-specific color mapping. The actual time bar denotes the length, relative to the other bars, of a fixed amount of time, e.g., 1 second ( *timeBar*). The overlap bar is white if the frames overlapped more than a threshold amount ( *threshAdjac*), and is otherwise black. The size of all three bars can also be adjusted ( *barSize*).

The final STL image is exported as an image file to the specified folder ( *path_out*). The image can also be viewed immediately ( *showSTL*).

### F. Path analysis method

If path analysis is enabled ( *doPath*), the STL toolbox uses a simple but efficient method to obtain x- and y-coordinates of the target at regular intervals ( *pathSampling*), which is often a higher sampling frequency than used for the STL image. In our example ( Figure 1B) we used 6 pps. These positions are plotted in a separate path image, which can either be overlaid on the STL image or the reference frame ( *pathBack*).

The path analysis method takes advantage of the same thresholds used in the STL algorithm to isolate the target and remove spatial and temporal noise. The coordinates of the target are determined by calculating the x- and y-coordinates for the center of the largest centroid, after the image has been intensity thresholded ( *threshTrim*). A minimum area for the largest centroid ( *areamin*) is also used to re-determine the start and end frames for the path analysis.

The obtained x- and y-coordinates for the target across all retained frames can be plotted over the STL or reference image. A color map is applied, along with the STL image, and the marker’s border and arrows can be modified in the configuration ( *pathCol*; usually black or white, depending on the background). The path image is saved in the same folder as the STL image ( *path_out*). Along with the x- and y-coordinates for each frame, two summary statistics are calculated: total path length and duration. If the pixels-to-meters conversion is specified ( *px2m*), coordinates and path length will be outputted in meters.

### G. Velocity-acceleration plot

Using the distances travelled between time points, as calculated for the path analysis, we can readily also calculate the instantaneous velocity and acceleration ( *doVel*). To reduce noise in these measures, a weighted average is taken across adjacent values ( *velSmooth*). The plot is saved in the same folder as the STL image ( *path_out*).

## Generalizability of the STL algorithm

 So far we have described the STL algorithm ( Figure 2) and presented images for one trial of a pigeon study ( Figure 1). To demonstrate the generalizability of the method, we tested it on videos of other animals.

**Figure 3.**
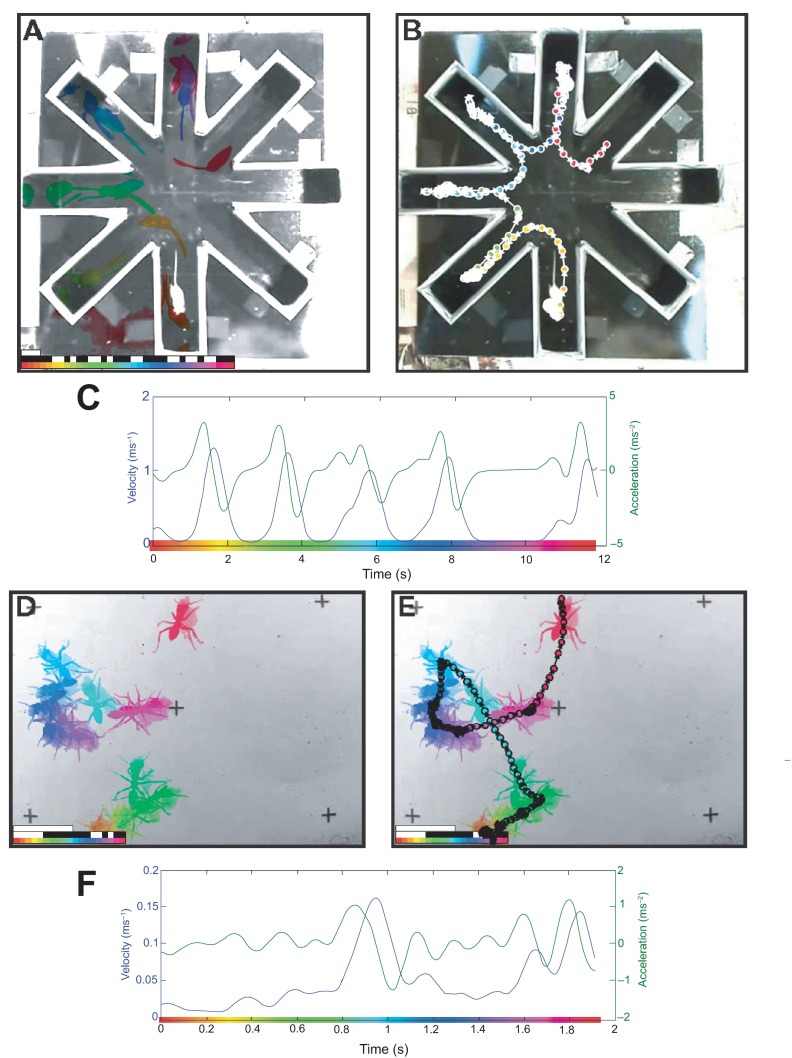
Application of the STL algorithm to videos of other animals. ( **A**) STL image of a mouse in a radial arm maze (available from http://www.youtube.com/watch?v=y7zQgz0vmWo, with permission of Anže Starič (University of Ljubljana)), sampled at 3 pps. First bar in bottom left corresponds to 1 second; second bar illustrates which frames highly overlapped with adjacent frames; third bar shows time-color mapping used. ( **B**) Path of same movement data as shown in panel **A**, overlaid on the reference frame, sampled at 30 pps. ( **C**) Velocity-acceleration plot of same movement data as panels **A** and **B**. ( **D**) STL image of an ant in an open environment, sampled at 10 pps (after adjusting for use of high-speed camera; available from http://www.youtube.com/watch?v=u7LaPjMtmYM with permission of Antoine Wystrach, Paul Graham, and Andrew Philippides (University of Sussex)). First bar in bottom left corresponds to 10 seconds; second bar illustrates which frames highly overlapped with adjacent frames; third bar shows time-color mapping used. ( **E**) Path of same movement data as panel **D**, overlaid on the STL image, sampled at 100 pps. ( **F**) Velocity-acceleration plot of same movement data as panels **D** and **E**.

 The first video, of a mouse in a radial-arm maze ( http://www.youtube.com/watch?v=y7zQgz0vmWo), was downloaded as a MPEG-4 file from YouTube and converted to an uncompressed AVI with MPEG Streamclip. We cropped the video to isolate the maze. As the video represented multiple trials, we chose a video segment from after the mouse had been trained, spanning from 1 min 46 sec to 1 min 59 sec; this temporal trimming was done through the STL toolbox by specifying the start and end frames (3178 and 3568, respectively). Several settings were modified to suit the video, such as the smoothing kernel size, color map cycles, and the target being lighter than the background. We sampled the mouse’s position at 3 pps for the STL image and 30 pps for the path analysis. We plotted the path over the reference frame. The resulting images are presented in Figures 3A–3C.

 The second video was of an ant in a simple open environment demonstrating scanning behaviour, where the ant is searching for visual landmarks ( http://www.youtube.com/watch?v=u7LaPjMtmYM). The video was also downloaded from YouTube and converted. Note that this video was recorded using a high-speed camera and had been slowed down by a factor of 10 (as stated in the video’s description). Settings were customized for differences in the video resolution and speed, as well as target size. Here we sampled the ant’s position at 10 pps, for the STL image and 100 pps for the path image. The resulting images are presented in Figures 3D–3F.

## Results and discussion

Here we presented a novel method of visualizing and quantifying animal movement from pre-recorded videos acquired with standard video equipment. The STL images accurately summarize an animal’s position at a given time, within a single two-dimensional image representation, and allow researchers to observe movement patterns without needing to watch full videos for every trial. We incorporated a simple but efficient path analysis method into the algorithm to quantify properties of the movement, including instantaneous velocity and acceleration. The STL toolbox implementing the STL algorithm in MATLAB is available freely from the authors. (For an introductory guide to MATLAB, see Madan, 2014).

As the path analysis method implemented in the STL toolbox is fairly simple, it has a few limitations: the method can only be used for a single target and it cannot correct for partially occluded targets or lens distortions. Several methods could be incorporated to allow for the tracking of multiple targets, such as placing unique markers on each target (e.g., Sakiyama *et al.*, 2006), identifying separable targets and calculating movement vectors or “limited-radius” searches for each (e.g., Perner, 2001; Tort *et al.*, 2006; Xu *et al.*, 2009), using shape templates (e.g., Kalafatić, 2003; Xu *et al.*, 2009), or using a particle-based approach (e.g., Khan *et al.*, 2006; Tweed & Calway, 2002). Future versions could use methods to correct for occlusions (e.g., Perner, 2001), which can include video artifacts such as timestamps embedded in the video (as in Figure 1). Estimates of path length may also be affected by lens distortions, e.g., if a fish-eye lens was used. These distortions can be corrected by combining manually-acquired known distances (i.e., a calibration grid) with the observed video data. ( Lind *et al.*, 2005) provide equations to compensate for lens distortions. Nonetheless, the path analysis method implemented here efficiently tracks a single target and requires no markers or shape templates.

Other fields have also demonstrated interest in movement-tracking methods. Most notably, many papers outlining methods for tracking movements have been published in the *Journal of Neuroscience Methods*, driven by interest in how neurological lesions or pharmacological manipulations influence movement. Our methods offer a simple, readily-available tool to complement existing techniques. These methods may also prove useful in other domains such as tracking humans from stationary surveillance cameras (e.g., Buono, 2011) or tracking vehicles over large areas (e.g., van Dommelen *et al.*, 2013).

## Data and software availability

### Data

figshare: Data File 1. Raw video used in Figure 1 and Figure 2. doi: http://dx.doi.org/10.6084/m9.figshare.900359 ( Madan & Spetch, 2014).

Copies of the YouTube videos have been deposited with *F1000Research* for archival purposes. Should the videos no longer be available from the respective YouTube links provided in the article, please contact *F1000Research*.

### Software

ZENODO: Spectral time-lapse (STL) Toolbox. doi: 10.5281/zenodo.7663 ( Madan & Spetch, 2014).

## Supplementary materials

Sample text feedback from running the STL toolbox (stltool) on video file '' **S−Video_20110718_1132.avi**'', the video of the single trial from the pigeon study. The outputted images are shown in Figure 1. (Output is text wrapped to fit on page.)

1   >> data = stltool(
                    'S−Video_20110718_1132.avi'
                    );
 2	
 3   Processing video file "S−Video_20110718_1132.avi"
 4   Reading from raw video (46 Frames)
 5   ..............................................
 6   Video is being sampled at one position per 1.00 seconds (1.0 pps)
 7   Checking frames for motion (46 Frames)
 8   ..............................................
 9   Colorizing frames (25 Frames)
10   .........................
11   Calculating spectral timelapse (STL) image
12   STL generated ("STL_S−Video_20110718_1132.tif")
13   STL summarizes 25.02 seconds of video
14   Processing video file "S−Video_20110718_1132.avi"
15   Reading from raw video (228 Frames)
16   ...................................................................
17   ...................................................................
18   ...................................................................
19   ...........................
20   Video is being sampled at one position per 0.20 seconds (5.0 pps)
21   Checking frames for motion (228 Frames)
22   ...................................................................
23   ...................................................................
24   ...................................................................
25   .............................
26   Detecting path
27   Path calculated ("STLpath_S−Video_20110718_1132.tif")
28   Total path length measured at 5.5423 m
29   Total path took 24.22 s
30   Velocity−acceleration plot generated
31   ("STLvel_S−Video_20110718_1132.pdf")

     Data output from same example.

1   >> data
 2   data =
 3		   config: [1x1 struct]
 4		    fname: 
                    'S−Video_20110718_1132.avi'

 5	   framesPathKept: 121
 6      framesPathSampled: 228
 7	    framesSTLKept: 25
 8       framesSTLSampled: 46
 9	       pathLength: 5.5423
10	         pathTime: 24.2239
11	          ppsPath: 4.9951
12		   ppsSTL: 0.9990
13	          trackXY: [121x2 double]
14	           velAcc: [1x118 double]
15	           velVel: [1x119 double]
16	        vidCDepth: 1
17	           vidFPS: 29.9704
18	        vidHeight: 480
19	         vidWidth: 640

     Legend of the outputted data's structure.

1		   config: backup of all config settings
 2		           (from config.m and configCustom.m)
 3		    fname: file name of video
 4	   framesPathKept: number of frames retained in path analysis
 5      framesPathSampled: total number of frames sampled for path analysis
 6		           (framesPathKept will always be a subset of this)
 7	    framesSTLKept: number of frames retained in STL image
 8       framesSTLSampled: total number of frames sampled for path analysis
 9		           (framesSTLKept will always be a subset of this)
10	       pathLength: total length of path (in pixels or meters)
11	         pathTime: total duration of path (in seconds)
12	          ppsPath: positions−per−second for path analysis
13		   ppsSTL: positions−per−second for STL image
14	          trackXY: x– and y−coordinates of path at each
15		           sampling point (in pixels or meters)
16	           velAcc: instantaneous acceleration
17		           (from velocity−acceleration plot)
18	           velVel: instantaneous velocity
19		           (from velocity−acceleration plot)
20	        vidCDepth: color depth of video
21		           (1 = grayscale; 3 = color [rgb])
22	           vidFPS: frames−per−second of video
23	        vidHeight: height of video
24	         vidWidth: width of video

     Example configuration settings code (config.m).

 1
                       %  Config settings for spectral time−lapse generation code (stltool)

  2
                       %  Written by Christopher R Madan

  3
                       %  Last edited 20131102 [CRM]

  4
                       %  Requires Statistics Toolbox (nanmean,normpdf)

  5
                       %  Requires Image Processing Toolbox (imresize,regionprops)

  6	
  7
                       %% general settings

  8
                       %  debug on? (activates 'interactive' mode at end of STL code)

  9   debugOn	        =  0;
 10
                       %  is the target lighter or darker than the background area?

 11
                       %  set to 1 for lighter, −1 for darker

 12   target	        =  −1;
 13
                       %  plot position from every i−th frame

 14
                       %  most videos are at 30 frames per second (30 Hz; NTSC)

 15   sampling	        =  30;
 16
                       %  video speed

 17
                       %  has the video frame rate been adjusted relative to the original

 18
                       %  recording?

 19
                       %  set to 1 if not

 20
                       %  set to .1 if used high−speed camera and slowed down by 10x

 21   videospeed	=  1;
 22
                       %  threshold for detecting change in frame

 23
                       %  if this is too low, there will be lots of 'speckle' (random noise)

 24
                       %  if this is too high, too few/no usable frames will be detected

 25   threshMask	=  50;
 26
                       %  block size of legend bars (height/width of each block, in px)

 27   barSize           =  8;
 28
                       %  length of time bar (in seconds)

 29   timeBar	        =  1;
 30
                       %  display STL image? (will be saved regardless)

 31   showSTL	        =  0;
 32
                       %  paths for input raw videos and output ot STL images

 33   path_raw	        =  
                    '../raw/'
                    ;
 34   path_out	        =  
                    '../output/'
                    ;
 35
 36
 37
                       %% frame range

 38
                       %  starting frame, used to manually remove the first i frames

 39
                       %  must be in quotes

 40   startFrame	=  
                    '1'
                    ;
 41
                       %  last frame, use 'lastFrame' for the last frame of the video

 42
                       %  must be in quotes

 43   endFrame	        =  
                    'lastFrame'
                    ;
 44
                       %  reference frame for subtraction (usually '1' or 'end')

 45
                       %  must be in quotes

 46
                       %  use 'move' for a moving average, can be a bit slow

 47   refFrame	        =  
                    'end'
                    ;
 48
                       %  if using moving average, how should frames be weighted

 49
                       %  (temporal smoothing)

 50
                       %  list of values should be odd in length

 51
                       %  middle value should be 0, so weight for 'current' frame is 0

 52
                       %  absolute values don't matter, will be normalized to sum to 1

 53   refSmooth         =  [ 4:–1:1 repmat(0,1,5) 1:1:4 ];
 54
 55
 56
                       %% frame spatial (masking)

 57
                       %  mask out a region of the frame if desired

 58   doMask	        =  1;
 59
                       %  if using doMask, specify mask filename (within path_raw)

 60   maskName	        =  
                    'mask.tif'
                    ;
 61
                       %  auto−mask white?

 62
                       %  E.g., if there was a hard−coded timestamp

 63   cleanWhite        =  1;
 64
                       %  lower end of what to trim as 'white'

 65   white             =  120;
 66
 67
 68
 69
                       %% frame temporal settings (trimming, change detection)

 70
                       %  disable auto−trimming of start and end frames

 71   disableTrim	=  0;
 72
                       %  threshold for automatically trimming start and end frames

 73
                       %  checks for differences between start/end frames and reference frame

 74
                       %  if there little difference, removes the frames

 75
                       %  (proportion of total frame)

 76   threshTrim	=  .004;
 77
                       %  threshold for detecting changes between adjacent frames

 78
                       %  used for the white/black bar to detect differences between adjacent

 79
                       %  frames

 80   threshAdjac	=  0.4;
 81	
 82	
 83
                       %% STL colorization settings

 84
                       %  set color map

 85
                       %  'hsv' − recommended, one color cycle

 86
                       %  'dhsv' − custom colormap for two cycles of hsv

 87
                       %  (dhsv = double hsv)

 88   cmap	        =  
                    'dhsv'
                    ;
 89
                       %  increase brightness of reference image (refFrame) by x

 90   oversatRef	=  2;
 91
                       %  increase saturation of colorized frames by x

 92   oversatCol	=  20;
 93
                       %  smoothing kernel range

 94
                       %  improves detection of change, reduces effects of random noise

 95   smooth	        =  −1:1; 
 96	
 97	
 98
                       %% path analysis settings

 99
                       %  calculate path image?

100   doPath	        =  1;
101
                       %  background image for path

102
                       %  'stl' or 'ref'

103   pathBack	        =  
                    'stl'
                    ;
104
                       %  color for arrows and marker circles

105
                       %  usually 'k' or 'w' (black or white)

106   pathCol	        =  
                    'k'
                    ;
107
                       %  different sampling rate for path analysis?

108
                       %  for same rate just type: 0

109
                       %  for double the STL sampling frequency: sampling/2

110   pathSampling	=  30/5;
111
                       %  minimum area for tracked

112
                       %  set to 0 for no minimum

113   areamin		=  200;
114
                       %  path output units

115
                       %  how many px in a meter

116
                       %  set to 0 to report as pixels

117   px2m	        =  0;
118	
119	
120
                       %% velocity−acceleration plot settings

121
                       %  calculate velocity−acceleration plot?

122   doVel	        =  1;
123
                       %  smooth instantaneous velocity/arousal with a kernel

124   velSmooth	        =  −3:.2:3;

     Example custom configuration code (configCustom.m).

1  
                    % put custom settings for specific videos here

 2	
 3  
                    if
                     strcmp(fname,
                    'ant.avi'
                    )
 4	threshMask	=  80;
 5	cmap	        =  
                    'hsv'
                    ;
 6	sampling	=  30;
 7	pathSampling	=  3;
 8	px2m	        =  100/(5/393);    
                    % 393 px   == 5 cm

 9	videospeed	=  .1;
10	timeBar	        =  1;
11	doMask	        =  0;
12	cleanWhite	=  0;
13  
                    elseif
                     strcmp(fname,
                    'radial.avi'
                    )
14	target	        =  1;
15	cmap	        =  
                    'hsv'
                    ;
16	startFrame	=  
                    '3178'
                    ;
17	endFrame	=  
                    '3530'
                    ;
18	sampling	=  10;
19	refFrame	=  
                    '1'
                    ;
20	smooth	        =  –2:2:2;
21	threshMask	=  30;
22	pathSampling	=  1;
23	pathBack	=  
                    'ref'
                    ;
24	pathCol	        =  
                    'w'
                    ;
25	px2m	        =  100/(100/330.5); 
                    % 330.5 px   == 100 cm

26	timeBar	        =  1;
27	doMask	        =  0;
28	cleanWhite	=  0;
29  
                    elseif
                     strfind(fname,
                    'S–Video'
                    )
30	timeBar	        =  10;
31	px2m	        =  100/(32/68.56); 
                    % 68.56 px   == 32 cm

32  
                    end
