# Supplementary material for: Visualizing and quantifying movement from pre-recorded videos: The spectral time-lapse (STL) algorithm
Source: F1000Res. 2014 Jan 21;3:19. [Version 1] doi: 10.12688/f1000research.3-19.v1 (PMC4038320; doi:10.12688/f1000research.3-19.v1)
Supplement: Peer review history

# Peer review: Referee response for version 1

**Published:** 2014-03-24

**Authors:** Michael Mangan

**Affiliations:** 1 Neuroinformatics and Computational Neuroscience, University of Edinburgh, Edinburgh, Scotland, UK

**Overview**

This paper describes a MatLab toolbox for tracking single animals in video data, quantifying basic movement properties (path-length, velocity profile, etc), and then displaying overall movement pattern in a single figure using colours to encode animal position at different times. 

The animal tracker presented comprises an elementary background subtraction approach augmented with masking, thresholding, and filtering methods to extract the location of the animal. As the parameters for these techniques are manually defined, and then held constant across the video, this method seems best suited to laboratory recordings where the environment changes little. For outdoor recordings, where there are likely illumination changes, occlusions etc a more robust tracking method is required e.g. TLD tracker (Z. Kalal, K. Mikolajczyk, and J. Matas, “ Tracking-Learning-Detection,” *Pattern Analysis and Machine Intelligence,* 2011). 

The authors summarise the movement of the animal in a "spectral-time-lapse" image. The STL overlays the animal position, colour coded with respect to time, on a reference image. I believe the primary benefit of this method is rapid visualisation of the position and orientation of the animal across its path. Although personally, I do not see the benefit of displaying the entire animal shape - I deduced more from the higher resolution track shown in Fig 1B for example. I also suspect that on longer or overlapping paths the STL would become cluttered and confusing.

The techniques used are mostly technically sound (see specific comments below). I foresee this work being of some use to behavioural researchers tracking animals in laboratory settings. Specifically: as the code is available as a Matlab toolbox, it can be easily installed, the parameters tuned for the specific case, and data visualised easily.

**Specific Comments**

- The authors describe a "moving average" reference frame, but do not describe how this is generated exactly. I think expanding upon this point to make it clear to a reader is important.
- The 3rd step in the Pre-processing algorithm inverts the negative pixel intensities. I think this step could be omitted by taking either a sum-squared or root-mean-square difference between reference and current frame in step 1.
- In the 4th step in the Pre-processing algorithm it is stated that the masking method " *is useful if timestamp or overlay is hard-coded in the video*". I would have thought this would be removed in the background subtraction step as it is constant across frames?
- The mouse data tracks the animal from 1 min 46 seconds until 1 min 59 seconds.  Was there a reason for using this specific portion of the video?

I have read this submission. I believe that I have an appropriate level of expertise to confirm that it is of an acceptable scientific standard.

# Peer review: Referee response for version 1

**Published:** 2014-02-27

**Authors:** Suzette Astley

**Affiliations:** 1 Department of Psychology, Cornell College, Mount Vernon, IA, USA

In this article, Madan and Spetch describe a tool for monitoring animal movement in an open field that will be extremely useful to many researchers. Not only does the software monitor the animal's path through the environment, it also offers time-based information, and can provide information about velocity and acceleration of the movement over time. MATLAB software is in wide use in behavioral research, and Madan and Spetch have made the STL algorithm freely available in MATLAB. This is the sort of new tool that can lead researchers to ask new questions and to look in new directions to answer current questions. This algorithm will undoubtedly make a significant contribution to our understanding of animal behavior.

I have read this submission. I believe that I have an appropriate level of expertise to confirm that it is of an acceptable scientific standard.
